# The Antiviral Activity of Caprylic Monoglyceride against Porcine Reproductive and Respiratory Syndrome Virus In Vitro and In Vivo

**DOI:** 10.3390/molecules27217263

**Published:** 2022-10-26

**Authors:** Luyu Yang, Jianhua Wen, Yang Zhang, Zheyan Liu, Zhipeng Luo, Lei Xu, Siyuan Lai, Huaqiao Tang, Xiangang Sun, Youjun Hu, Ling Zhu, Zhiwen Xu

**Affiliations:** 1 College of Veterinary Medicine, Sichuan Agricultural University, Chengdu 611130, China; 2 Key Laboratory of Animal Diseases and Human Health of Sichuan Province, Sichuan Agricultural University, Chengdu 611130, China; 3 Innovation Centre of Guangdong Nuacid Biotechnology Co., Ltd., Qingyuan 511545, China

**Keywords:** PRRSV, antiviral, medium-chain fatty acids, caprylic monoglyceride, weaned piglets

## Abstract

Porcine reproductive and respiratory syndrome (PRRS) is a disease with a major economic impact in the global pig industry, and this study aims to identify potential anti-PRRSV drugs. We examined the cytotoxicity of four medium-chain fatty acids (MCFAs) (caprylic, caprylic monoglyceride, decanoic monoglyceride, and monolaurin) and their inhibition rate against PRRSV. Then the MCFAs with the best anti-PRRSV effect in in vitro assays were selected for subsequent in vivo experiments. Potential anti-PRRSV drugs were evaluated by viral load assay, pathological assay, and cytokine level determination. The results showed that caprylic monoglyceride (CMG) was the least toxic to cells of the four MCFAs, while it had the highest PRRSV inhibition rate. Then the animals were divided into a low-CMG group, a medium-CMG group, and a high-CMG group to conduct the in vivo evaluation. The results indicated that piglets treated with higher concentrations of caprylic monoglyceride were associated with lower mortality and lower viral load after PRRSV infection (*p* < 0.05). The pulmonary pathology of the piglets also improved after CMG treatment. The levels of pro-inflammatory cytokines (IL-6, IL-8, IL-1β, IFN-γ, TNF-α) were significantly downregulated, and the levels of anti-inflammatory cytokine (IL-10) were significantly upregulated in the CMG-treated piglets compared to the positive control group (*p* < 0.05). Taken together, the present study revealed for the first time that caprylic monoglyceride has strong antiviral activity against PRRSV in vitro and in vivo, suggesting that caprylic monoglyceride could potentially be used as a drug to treat PRRS infection.

## 1. Introduction

Porcine reproductive and respiratory syndrome (PRRS) is a highly contagious disease caused by porcine reproductive and respiratory syndrome virus (PRRSV), which was first identified in the United States in 1987 and is an economically impactful disease in the global pig industry [1,2]. To date, PRRSV is known specifically to infect pigs, and there are no data to suggest that other species are susceptible to this pathogen [3]. PRRSV replicates primarily in porcine alveolar macrophages, and the clinical signs of PRRSV infection vary among pigs at different ages. Adult sows infected with PRRSV can develop reproductive disorder syndromes such as stillbirth, mummified fetuses, early embryonic death, and sterility, while respiratory syndromes are more common in PRRSV-infected piglets [4]. PRRSV infection also increases the risk of coinfection with other pathogens because it compromises the host’s immune system. To date, investigations have been carried out to examine pathogenesis, the host immune response to viral infection, and how PRRSV survives in the host. PRRSV is internalized into host cells by the interaction between PRRSV proteins and cellular receptors. When the virus invades the cells, the host antiviral immune system is quickly activated to suppress the replication of the virus. To retain fitness and host adaptation, various viruses have evolved multiple complex strategies to manipulate the host machine and circumvent the host antiviral responses [5]. PRRSV vaccines have been developed, including inactive viruses, live modified viruses, live attenuated vaccines, DNA vaccines, and immunoadjuvant vaccines [6]. However, there is some controversy regarding their efficacy. In addition, the high variability of field viruses and the ongoing emergence of new variants pose a challenge for PRRSV vaccine development [7]. Therefore, the discovery of potential anti-PRRSV drugs continues to be a high priority in the global pig industry [8].

Medium-chain fatty acids (MCFAs) are fatty acids which consist of 8–10 carbon atoms, are found in small amounts in nature, and are mainly derived from milk, breast milk, palm kernel oil, and coconut oil [9]. MCFAs have antimicrobial properties and, depending on their dose, have some effect on the productive performance of animals [10]. For example, MCFAs have been shown to reduce the risk of porcine epidemic diarrhea virus (PEDV) transmission through feed and ingredients [11,12,13]. MCFAs can influence the growth performance of animals and can function as ready-to-use energy substrates, gastrointestinal morphological modifiers, and antimicrobial compounds [14,15]. Tran et al. [16] showed that MCFAs can be added to feed to suppress African swine fever (ASF) virus transmission. Since MCFAs have indicated potential antiviral and antimicrobial effects, it is important to study and evaluate the therapeutic effects of MCFAs against PRRSV to widen their application value as feed additives.

## 2. Results

### 2.1. Maximum Nontoxic Concentration Determination

**Figure 1:**
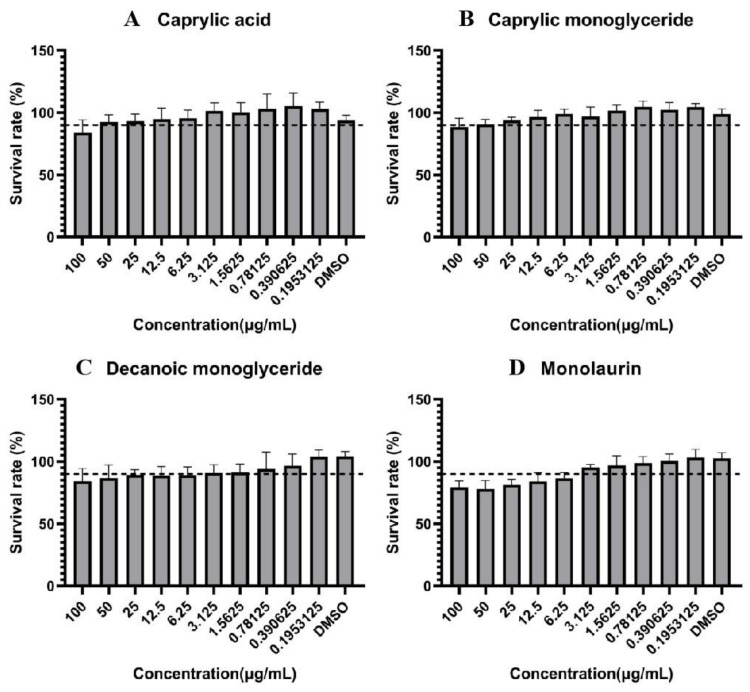
Survival rate of Marc-145 cells. (**A**): caprylic acid; (**B**): caprylic monoglyceride; (**C**): decanoic monoglyceride; (**D**): monolaurin. 90% survival rate as a safety scale (dashed line). The highest concentration at which cell survival rate exceeds 90% is the maximum nontoxic concentration (MNTC). Samples with solvent DMSO addition were used as a no-inhibition control.

We determined and calculated the survival rate of Marc-145 cells after incubation with different concentrations of MCFAs to determine the maximum nontoxic concentration (MNTC) of each MCFA. The results are shown in Figure 1. They indicate that Marc-145 cells tolerated caprylic acid and caprylic monoglyceride well with a MNTC of 50 μg/mL, while decanoic monoglyceride and monolaurin were toxic to cells with 1.5625 and 3.125 μg/mL of MNTC, respectively. The cell survival rates in the DMSO group were all above 90%, indicating that the toxicity of DMSO as a solvent to the cells was negligible.

### 2.2. Virus Inhibition Assay

**Figure 2:**
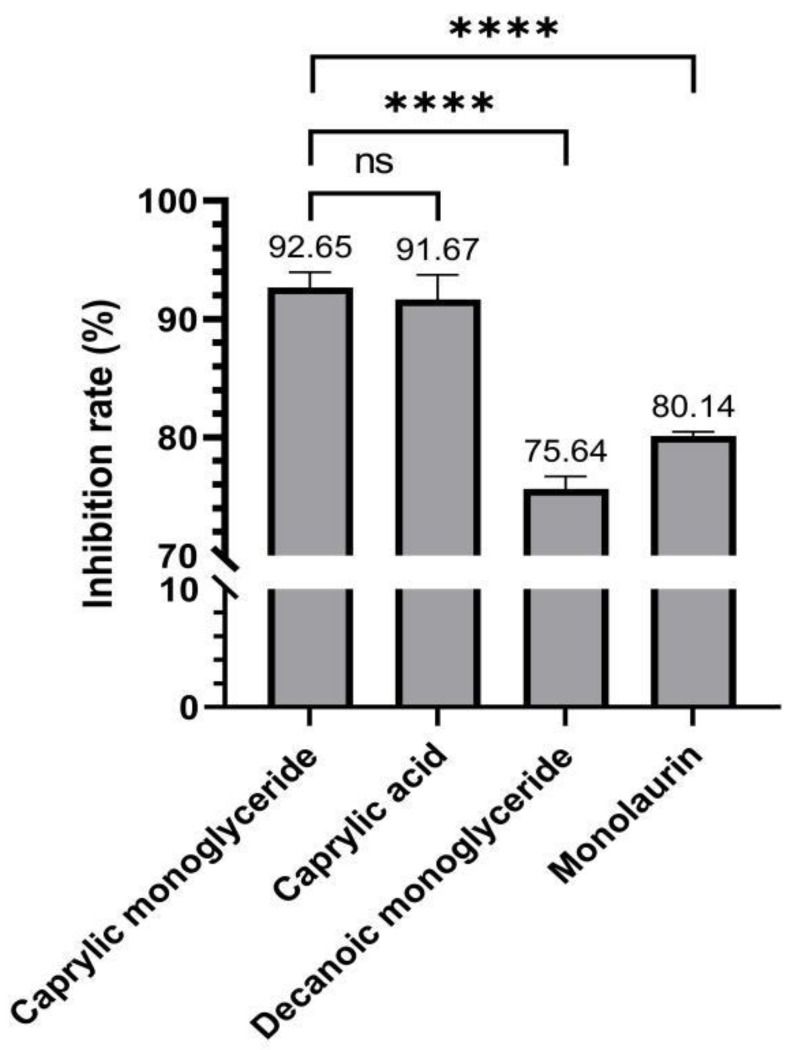
Inhibition rate to PRRSV of all four MCFAs. **** *p* < 0.0001, ns indicates no significant difference between the two groups.

Virus inhibition of four MCFAs at MNTC was measured by the CCK8 method. The results of the virus inhibition assay showed that all four MCFAs could inhibit the proliferation of PRRSV (Figure 2). Meanwhile, caprylic acid and caprylic monoglyceride showed higher inhibitory ability against PRRSV, both above 90%. Decanoic monoglyceride and monolaurin showed a significantly lower inhibitory effect on PRRSV than caprylic monoglyceride and caprylic acid (*p* < 0.0001). However, caprylic monoglyceride showed relatively higher inhibition to PRRSV than caprylic acid at the concentration of 100 mg/mL. Under sample-limited conditions, we selected caprylic monoglyceride for subsequent in vivo testing.

### 2.3. Observation and Evaluation of Clinical Signs

**Figure 3:**
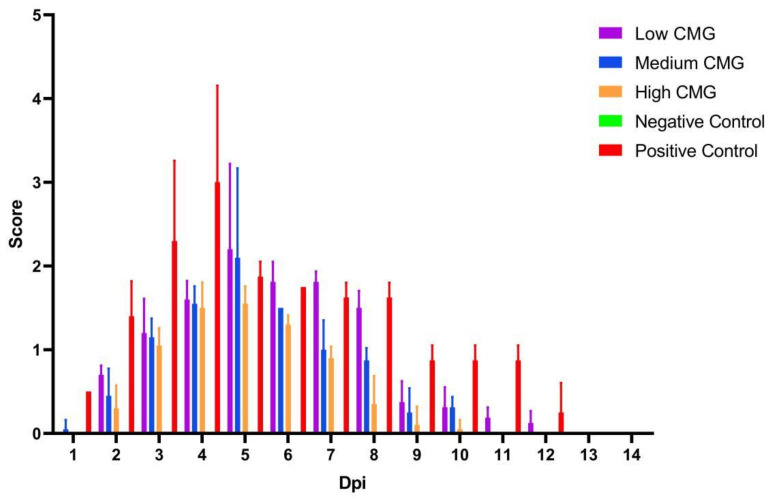
The clinical signs’ scores. Clinical signs, including parameters such as loss of appetite, respiratory distress, fever, and behavioral lethargy. Clinical parameters were scored using values 0–2 (none = 0; mild = 1; severe = 2), while survival was recorded in the same way with values 0–1 (survived = 0; died = 1). Based on the recorded parameters, the clinical score (CS) was calculated with the formula: CS = {[(numeric value of anorexia + numeric value of respiratory clinical signs + numeric value of fever + numeric value of lassitude)/4] + 4 × (numeric value of survival)}. The final average score of the five piglets in each group was taken. The more severe the clinical signs of the piglet, the higher the score.

The 25 piglets were randomly divided into five groups (negative control, positive control, high CMG, medium CMG, and low CMG), with five piglets in each group. Detailed grouping and dosage information are shown in Table 1. We observed and assessed the clinical signs of the piglets daily for a total of 14 days after completion of the infection and dosing steps (Figure 3). During the 14-day observation period, a total of five piglets died. On day 5, there was one death in the positive control group; on day 6, there were two deaths in the positive control group and one death each in the low-CMG and medium-CMG groups. After the thirteenth day, the piglets basically no longer showed clinical signs. During the experiment, the negative control group had no obvious clinical signs from the first day to the fourteenth day, and there were no deaths in the high-CMG group or the negative control group. The piglets in the positive control and low-CMG groups showed severe respiratory clinical signs, high fever, and reduced food intake, while the piglets in the medium-CMG and high-CMG groups showed mild clinical signs.

### 2.4. PRRSV Load in Swabs and Tissues

**Figure 4:**
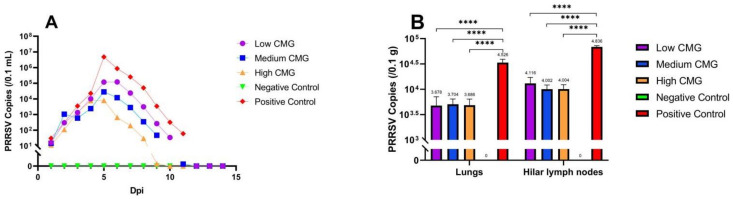
Viral load detection in piglets infected with PRRSV. (**A**): Changes in viral load in nasal swabs of the five groups of piglets for 14 consecutive days; (**B**): Comparison of viral loads in the lungs and hilar lymph nodes of the five groups of piglets. **** *p* < 0.0001.

We measured PRRSV load in lung tissues and nasal swabs by RT-qPCR. The viral load in the nasal swabs of the piglets showed an increasing trend in the first five days, peaked at day 5, and then showed a decreasing trend. The virus could not be detected on day 12 after infection. Meanwhile, the viral loads in the lungs and hilar lymph nodes of the piglets in the three groups after administration were lower than those in the positive control group (*p* < 0.0001), and there was no virus in the lungs of the piglets in the negative control group, indicating that caprylic monoglyceride reduced the viral load in the lungs and hilar lymph nodes of the piglets infected with PRRSV in a dose-dependent manner (Figure 4).

### 2.5. Autopsy Analysis and Histopathological Examination

**Figure 5:**
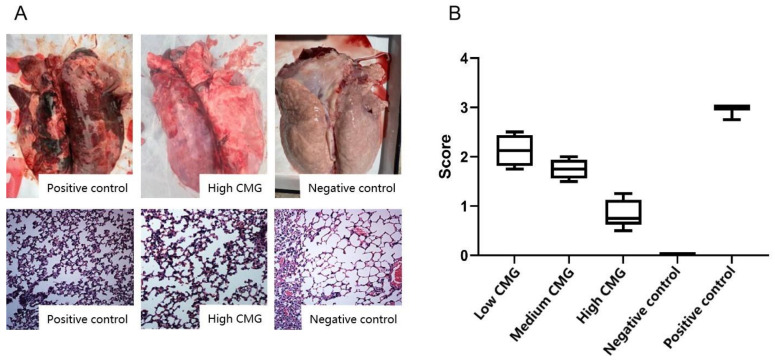
Pathological testing and evaluation. (**A**): lung autopsy analysis and lung histopathological examination results. (**B**): comparison of pathology scores after lung dissection in five groups of piglets.

After 14 days of observation, all the piglets were euthanized and their lungs were immediately removed for pathological examination and assessment. As shown in Figure 5B, the pathological scores of the caprylic monoglyceride-treated samples were lower than the positive control group, with the most significant reduction in pathological scores in the high CMG group. Therefore, we selected the positive control group, the high-CMG group, and the negative control group for comparison of lung autopsy results and pathological sections (Figure 5A). The necropsy analysis showed that the positive control group had the most severe lung lesions, while the high CMG and negative control groups had no significant lung lesions. The lungs of the piglets in the positive control group showed severe interstitial widening with pulmonary congestion, hemorrhage, oedema, and pulmonary oedema, whereas the lungs of the piglets in the high-CMG group showed slight pathological changes in congestion, hemorrhage, and interstitial widening. Pathological section observations showed that both the positive control group and the high-CMG group had a large number of inflammatory cells and erythrocyte infiltration, alveolar epithelial cell detachment, and necrosis, while there were no pathological changes in the negative control group.

### 2.6. Cytokine Assay

**Figure 6:**
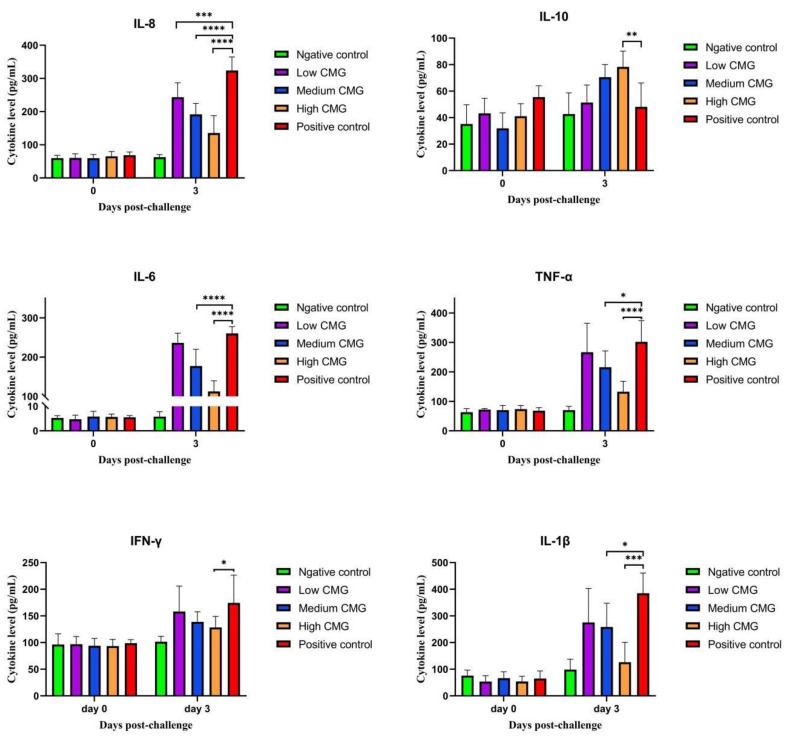
Effects of caprylic monoglyceride on cytokine (IL-6, IL-8, IL-IL-0, IL-1β, IFN-γ, TNF-α) levels in blood. Shows the changes in cytokine levels at 0 dpi and 3 dpi and a comparison of cytokine levels among the five groups of piglets at 3 dpi. Statistical significance is denoted by * *p* < 0.05, ** *p* < 0.01, *** *p* < 0.001, **** *p* < 0.0001.

Venous blood was collected from the piglets at 0 dpi and 3 dpi respectively, and the levels of the cytokines (IL-6, IL-8, IL-IL-0, IL-1β, IFN-γ, TNF-α) were measured by ELISA. As shown in Figure 6, pro-inflammatory cytokines significantly increased after PRRSV infection. Caprylic monoglyceride showed good anti-inflammatory activity. High doses of caprylic monoglyceride significantly decreased the levels of IL-1β, IFN-γ, and TNF-α and were not significantly different compared to the negative control group (*p* > 0.05). Caprylic monoglyceride also significantly increased the levels of IL-10 in a dose-dependent manner.

## 3. Discussion

The pig industry has suffered huge economic losses due to PRRSV. Current vaccines do not provide complete protection, the virus mutates rapidly, and researchers continue to discover new variants [17]. Antiviral therapy may be one of the main research directions for preventing PRRSV infection and reducing PRRSV viral load in the future [18]. MCFAs are healthy food components with about half the carbon chain length of long-chain fatty acids. The caprylic, caprylic monoglyceride, decanoic monoglyceride, and monolaurin mentioned in this study are all MCFAs. MCFAs have inhibitory effects on PEDV [19] and ASFV [20]. Caprylic acid inhibits ASFV under liquid conditions, which may help to inhibit the disease transmission. Researchers using caprylic acid significantly reduced bacterial translocation, enhanced antimicrobial activity, and significantly increased secretion of pBD-1 and pBD-2 [20]. A monolaurin mixture mitigated and prevented the transmission of contaminated feed to piglets [21]. However, it has not been reported whether MCFAs have an inhibitory effect on PRRSV. In the present study, we demonstrate that caprylic monoglyceride showed strong anti-PRRSV activity in vitro and in vivo. This suggests that caprylic monoglyceride could be applied in broad-spectrum anti-PRRSV medications.

MNTC is an important indicator in antiviral therapy studies [22,23]. In this study, we measured the MNTC of four MCFAs on cells, and the results showed that both caprylic acid and caprylic monoglyceride were less toxic to cells than decanoic monoglyceride and monolaurin. Caprylic monoglyceride showed better inhibition of PRRSV than caprylic acid at a concentration of 100 mg/mL and was selected for subsequent in vivo testing. Although caprylic monoglyceride inhibited PRRSV slightly more than caprylic acid, there was no significant difference between them, so the anti-PRRSV effect of caprylic acid in vivo should also be verified in future studies. At the same time, we wonder whether the combined administration of two or more medium-chain fatty acids could work better.

In vivo tests alone are imperfect and require observation of clinical signs, detection of viral load in the infected piglets, and histopathological examination to assess the efficacy of caprylic monoglyceride against PRRSV [24,25]. In this study, clinical signs were most evident in the weaned piglets on days 5 and 6 to confirm the effect of caprylic monoglyceride, and mortality was higher on those two days. The viral load of the PRRSV showed an increasing trend in the first four days after infection and administration, peaked at days 5 and 6, and then showed a decreasing trend, which coincided with the results of the clinical symptom scores. On the fifth day, the viral load was inversely proportional to the caprylic monoglyceride dose, i.e., the higher the caprylic monoglyceride dose, the lower the viral load. During the acute phase of PRRSV infection, the lung is the preferred location for viral replication because porcine alveolar macrophages are the primary target cells [26,27]. In the later stages of infection, virus replication is mainly confined to lymphoid organs, such as tonsils and lymph nodes, where the virus can persist for several months, and the continued replication of the virus in the lymph nodes contributes to efficient virus transmission [28,29]. Fourteen days after the caprylic monoglyceride administration, the viral loads in the lungs and lymph nodes of the weaned piglets were significantly lower than those in the positive control group. It is speculated that caprylic monoglyceride may play a role in the replication phase of the virus, and the exact mechanism is subject to further study.

In this study, the pathological status of the lungs of the weaned piglets after the caprylic monoglyceride administration improved, but the pathological sections of the piglets in the high-CMG group still showed a large infiltration of inflammatory cells and erythrocytes, probably because the drug started to work only on the fifth day, before which the lungs of the PRRSV-infected piglets suffered some pathological damage and the tissue sites selected for section preparation had not been repaired. In subsequent studies, subgroups should also be added to the samples for pathological testing on day 5 after the drug administration.

The inflammatory response is the first line of defense against the spread of viral infections. However, uncontrolled inflammation usually leads to severe damage to the host [30]. IL-6, IL-8, IL-1β, TNF-α, and IFN-γ are pro-inflammatory cytokines involved in promoting the acute inflammatory response and defending against infection. PRRSV infection triggers the up-regulated release of IL-1β, IL-6, IL-8, and TNF-α [31,32]. IL-10 is an anti-inflammatory cytokine that is an indicator of the body’s anti-inflammatory response [33]. After caprylic monoglyceride treatment, the levels of pro-inflammatory cytokines were significantly lower and the levels of anti-inflammatory factors were higher in the caprylic monoglyceride–treated group compared to the positive control group. This suggested that caprylic monoglyceride moderates the PRRSV-induced inflammatory responses by decreasing mRNA expression of pro-inflammatory cytokines, thereby inhibiting viral replication and restoring the body to its normal direction to avoid severe inflammation. Yu et al. found that ginsenoside Rg1 inhibited PRRSV infection in vitro via the NF-κβ signaling pathway and was partially protective against HP-PRRSV in piglets. Their results showed that the expression levels of several pro-inflammatory factors, including IL-1β, IL-6, IL-8, and TNF-α, reduced significantly in Marc-145 cells and PAMs after Rg1 treatment following PRRSV infection. The results were consistent with the present study [34].

## 4. Materials and Methods

### 4.1. Preparation of the MCFAs

MCFAs, including caprylic, caprylic monoglyceride, decanoic monoglyceride, and monolaurin, were prepared by Guangdong Nuacid Biotechnology Co., Ltd. (Guangdong, China). The MCFA powders were dissolved to completion in dimethyl sulfoxide (DMSO) (Sigma, St. Louis, MO, USA) at 10 mg/mL as the stock solutions and stored at 4 °C.

### 4.2. Cells and Virus

Marc-145 cells were cultured in Dulbecco’s modified Eagle’s medium (DMEM) (Gibco, Grand Island, NY, USA) supplemented with 10% fatal bovine serum (GibcoTM, Thermo Scientific, Waltham, MA, USA), 1% penicillin/streptomycin (GibcoTM, Thermo Scientific), and 25 μg/mL of gentamicin (GibcoTM, Thermo Scientific) at 37 °C in 5% CO_2_ for 48 h to reach a confluence of 70–80%. PRRSV was cultivated in grown monolayer Marc-145 cells for 48 h at 37 °C in 5% CO_2_. The virus was harvested using three −80 °C freeze–thaw cycles followed by a 12,000 rpm centrifuging for 10 min at 4 °C. The supernatant was then collected and stored at −80 °C for further use. The cells and virus used in this experiment were stored and provided by the Animal Biotechnology Center, School of Veterinary Medicine, Sichuan Agricultural University.

### 4.3. In Vitro Cytotoxicity Assay

The stock solutions of the MCFAs were diluted to 11 concentrations from 0.1 mg/mL to 0.1 × 10^−10^ mg/mL by adding DMEM supplemented with 2% fetal bovine serum. Once the growth of Marc-145 cells covered 70–80% of the culture dish, the cells were exposed to different concentrations of MCFA solution and cultured at 5% CO_2_, 37 °C for 48 h. The cell viability was measured using a CCK8 kit (Beyotime Biotechnology Co., Ltd., Shanghai, China) according to the manufacturer’s instructions. The maximum non-toxic concentration (MNTC) of the MCFAs on Marc-145 was calculated.

### 4.4. Infection Inhibition Assay In Vitro

The Marc-145 cells were cultivated into 96-well plates and incubated to a monolayer at 37 °C in a 5% CO_2_ incubator. After incubation for 2 h in a MNTC MCFA, Marc-145 cells were exposed to a mixture of MNTC MCFA and 100 TCID50 PRRSV (100 μL/well) and then incubated for 1 h at 37 °C in 5% CO_2_. Then the supernatant was discarded and 100 μL MNTC MCFA solution was added to each corresponding well and incubated for 36–48 h at 37 °C in 5% CO_2_. The development of a cytopathic effect (CPE) in the PRRSV-positive control group was observed periodically, and the incubation was stopped immediately when any apparent CPE was observed in the negative control group. The supernatant was then collected, and the cell viability was measured using the CCK8 assay. The viral inhibition rate was calculated.

### 4.5. Anti-PRRSV Activity Evaluation In Vivo

Twenty-five 6-week-old piglets were obtained from a commercial farm in Chengdu, China. All the piglets were healthy and free from African swine fever virus (ASFV), PRRSV, pseudorabies virus (PRV), and swine influenza virus (SIV) (negative for antibodies by ELISA (Invitrogen™, Thermo fisher, Chengdu, China)and negative for antigens by RT-PCR). The piglets were housed in the experimental environment for three days to acclimatize them to the new environment. The 25 piglets were randomly divided into five groups (negative control, positive control, high-CMG, medium-CMG, and low-CMG, where CMG stands for caprylic monoglyceride), with five piglets in each group. Except for the negative control group, the piglets were infected with PRRSV using the nasal drip method. After 24 h of nasal drip, the success of PRRSV infection was detected by PCR, and the MCFA preparation was given orally to the three groups (high-CMG, medium- CMG, and low-CMG) of piglets once a day for three days. Detailed grouping and dosage information are shown in Table 1. The groups were kept in different but environmentally similar spaces to prevent mutual influence.

#### 4.5.1. Clinical Signs

Clinical signs, including parameters such as loss of appetite, respiratory distress, fever, and behavioral lethargy, were observed daily in each group, once a day by the same researcher, during which the rectal temperature of each piglet was measured. Clinical parameters were scored using values 0–2 (none = 0; mild = 1; severe = 2), while survival was recorded in the same way with values 0–1 (survived = 0; died = 1). On the basis of the recorded parameters, the clinical score (CS) was calculated with the formula: CS = {[(numeric value of anorexia + numeric value of respiratory clinical signs + numeric value of fever + numeric value of lassitude)/4] + 4 × (numeric value of survival)}, and the final average score of the five piglets in each group was taken [35]. In addition, nasal swabs and jugular venous blood were collected daily to measure the PRRSV load. When severe respiratory signs were observed or a piglet was in the near-death stage, euthanasia was performed immediately and then the piglet was dissected. The lungs were rapidly removed and fixed with 4% paraformaldehyde. On day 14, all the piglets were euthanized, followed by dissection; the lungs were immediately collected, fixed in 4% paraformaldehyde, and examined for pathological changes.

#### 4.5.2. Quantitative Detection of PRRSV Load in Tissues and Nose Swabs by RT-qPCR

Nasal swabs were collected daily from the piglets after they were infected with PRRSV. All the collected swabs were immersed in 1 mL PBS immediately and centrifuged at 12,000 r/min for 3 min. Lung and hilar lymph node tissues were ground into homogenates. Each homogenate was thoroughly mixed with 3 mL PBS and centrifuged at 12,000 r/min for 5 min. All supernatants were collected, and the total RNA was extracted using the Trizol method (Takara, Dalian, China). The cDNA was obtained by reverse transcription of RNA using the prime™ RT kit (Perfect Real Time) (Takara, Dalian, China). The cDNA was loaded into fluorescent quantitative PCR (SYBR Green, Takara) to detect the viral load of the PRRSV. The qPCR reaction was 10 μL: 5 μL SYBR Green Premix Ex-TaqⅡ, 0.5 μL upstream primers (primer F: AATTCCCGCACCTCGCGGAACTGT), 0.5 μL downstream primers (primer R: AGTTCCTGCACCGCGTAGAACTGT), 1 μL cDNA, and added ddH2O to 10 μL. The qPCR reaction conditions were as follows: performed at 95 °C pre-denaturation for 30 s, 95 °C pre-denaturation for 5 s, and 60 °C pre-denaturation for 30 s and 40 cycles. Melting curve: 95 °C, 0.5 °C rise at 65 °C to 95 °C/s.

#### 4.5.3. Histopathology

After being fixed with 4% paraformaldehyde, the lungs were dehydrated, cleared, embedded, sectioned, dewaxed, rehydrated, stained, and mounted to prepare the stained sections. Histopathological changes were observed under light microscopy and were photographed. Pathological changes were also scored with values 0–2 (none = 0; mild = 1, moderate = 2; severe = 3). The pathology score (PC) was calculated using the formula: PS = [(value of pulmonary inflammatory exudates + value of solid lung tissue + value of pulmonary passages + value of IPF)/4].

#### 4.5.4. Cytokine Level Determination

Venous blood was collected from the piglets at 0 dpi and 3 dpi, respectively, and the cytokines levels (including IL-6, IL-8, IL-10, IL-1β, IFN-γ, and TNF-α) were measured using ELISA kits. All the tests were performed as per the kit manuals.

### 4.6. Statistical Analyses

The results are expressed as mean ± standard deviation. Significant differences were determined in SPSS 20.0 using paired-samples *t*-tests or one-way analysis of variance followed by Duncan’s multiple range test. *p* < 0.05 indicates a statistically significant difference.

## 5. Conclusions

In conclusion, caprylic monoglyceride has strong anti-PRRSV activity in vitro. In vivo, caprylic monoglyceride reduced the viral load of PRRSV, along with a reduction in lung injury and inflammatory response. It is suggested that caprylic monoglyceride could be a potential drug for the treatment of PRRSV.

## Figures and Tables

**Table 1:** Piglet grouping, virus challenge, and administration information.

Group	*n*	PRRSV	Dosage of the Drug	Treatment Time
Negative control	5	/	/	Day 1, 2 and 3
Positive control	5	10^5^ TCID_50_/mL	/	
Low CMG	5	10^5^ TCID_50_/mL	0.5 g/kg	
Medium CMG	5	10^5^ TCID_50_/mL	1.0 g/kg	
High CMG	5	10^5^ TCID_50_/mL	2.0 g/kg	

Note: The oral dose of the drug was determined by the weight of the piglets and given once a day for three days starting 24 h after the piglets were infected with the virus. TCID_50_ = 10^−7^/mL virus stock solution. The attack doses were all 1 mL of 10^5^ TCID_50_/mL, equivalent to 10^−2^/mL virus stock solution.

## Data Availability

The datasets analyzed in this study are available from the corresponding author on reasonable request.

## Sample Availability

Samples of the caprylic monoglyceride and the PRRS virus are available from the correspondence author.
